# Molecular characterization, virulence and antimicrobial and biocidal susceptibility of selected bacteria isolated from the cloaca of nestling ospreys (*Pandion haliaetus*) from Mono Lake, California, USA

**DOI:** 10.1371/journal.pone.0311306

**Published:** 2024-09-27

**Authors:** Igor Loncaric, Michael P. Szostak, Adriana Cabal-Rosel, Olivia M. Grünzweil, Alina Riegelnegg, Dusan Misic, Elke Müller, Andrea T. Feßler, Sascha D. Braun, Stefan Schwarz, Stefan Monecke, Ralf Ehricht, Werner Ruppitsch, Joachim Spergser, Ashli Lewis, Peter H. Bloom, Miguel D. Saggese

**Affiliations:** 1 Institute of Microbiology, University of Veterinary Medicine Vienna, Vienna, Austria; 2 Austrian Agency for Health and Food Safety (AGES), Institute of Medical Microbiology and Hygiene, Vienna, Austria; 3 Department of Functional Food Products Development, Faculty of Biotechnology and Food Science, Wroclaw University of Environmental and Life Sciences, Wroclaw, Poland; 4 Leibniz Institute of Photonic Technology (IPHT), Jena, Germany; 5 InfectoGnostics Research Campus, Jena, Germany; 6 Centre of Infection Medicine, School of Veterinary Medicine, Institute of Microbiology and Epizootics, Freie Universität Berlin, Berlin, Germany; 7 Veterinary Centre for Resistance Research (TZR), School of Veterinary Medicine, Freie Universität Berlin, Berlin, Germany; 8 Institut für Medizinische Mikrobiologie und Hygiene, Universitätsklinik Dresden, Dresden, Germany; 9 Institute of Physical Chemistry, Friedrich Schiller University, Jena, Germany; 10 California State Parks, Grass Valley, CA, United States of America; 11 Bloom Research Inc, Santa Ana, CA, United States of America; 12 College of Veterinary Medicine, Western University of Health Sciences, Pomona, CA, United States of America; Universidad San Francisco de Quito, ECUADOR

## Abstract

In the present study, the presence of the Enterobacterales, *Staphylococcus* spp., *Mammaliicoccus* spp., and *Enterococcus* spp. in cloacal samples of nestling ospreys (*Pandion haliaetus*), a fish-eating specialist, from Mono Lake, California, USA was examined by a multiphasic approach, including antimicrobial and biocide susceptibility testing, genotyping, and whole genome sequencing of selected isolates. The most commonly detected species was *Escherichia coli*, followed by *Mammaliicoccus sciuri*, *Staphylococcus delphini*, *Enterococcus faecalis*, *Enterococcus faecium*, *Hafnia alvei*, *Klebsiella pneumoniae*, *Citrobacter braakii* and single isolates of *Edwardsiella tarda*, *Edwardsiella albertii*, *Klebsiella aerogenes*, *Plesiomonas shigelloides* and *Staphylococcus pseudintermedius*. Multi-drug resistance (MDR) was observed in two *E*. *coli* isolates and in an *Enterococcus faecium* isolate. The MDR *bla*_CTX-M-55_-positive *E*. *coli* belonged to the pandemic clone ST58. The results of the present study suggest that nestling ospreys are exposed to MDR bacteria, possibly through the ingestion of contaminated fish. Ospreys may be good biosentinels for the presence of these microorganisms and antibiotic resistance in the local environment and the risk for other wildlife, livestock and humans.

## Introduction

The osprey (*Pandion haliaetus*) is a large, specialized fish-eating bird of prey with a worldwide distribution [1]. The species is associated mainly with lakes, rivers, seacoasts, and other water bodies present in a wide range of habitats, where they can capture prey and breed [2]. With its unique features in its osteology, pelvic musculature, the distribution of feather tracts, and results of modern molecular genetics studies, the osprey is broadly accepted to be a unique, monotypic, and distinct taxon that differs from other diurnal birds of prey [3]. For more detailed information on the ecology and natural history of the species, see [2, 4].

The North American continental subspecies (*P*. *haliaetus carolinensis*) was severely affected by DDT and other organochlorine pesticides in the past century, as these pollutants interfere with calcium metabolism, causing eggshell thinning, embryo mortality, and as a result negatively impacted hatching and breeding success of birds [5, 6]. Between 1950 and 1970 several osprey populations in North America collapsed [2]. In California, ospreys were also declining since the early 1900s, due to habitat loss, shooting and contamination. In California, ospreys originally nested along the Pacific coastline [7–10] and along the major inland lakes [11]. By the late eighties, and once the osprey’s population started to recover as the result of the banning of DDT and other organochlorine pesticides, new areas started being colonized by the species, facilitated sometimes by the presence of new nesting sites such as anthropogenic structures, and/or food availability in the form of seeded fish in inland lakes [4, 12]. One of these recently colonized nesting areas is Mono Lake in California.

By 1985, ospreys were observed for the first-time nesting in the Mono Lake, an inland, alkaline, endorheic water body located in Mono County, eastern central California [13]. In this lake, the presence of calcium carbonate towers, locally called “tufas” that emerged as a result of the reduction in water levels provided new available nesting sites for the species. Ospreys build these nests on the top of the tufa, which are frequently located offshore and surrounded by water, making access to the nest to mammalian predators almost impossible. As a result, breeding success in this area is usually high [12], Bloom unpublished data). In 2021 and 2022, approximately 15–17 pairs were nesting in this lake (A. Lewis pers. com, Bloom unpublished data). This unique and relatively small osprey population relies on fish captured in rivers and lakes located nearby, usually between <1 km to 11 km, as Mono Lake does not support any fish population due to its hypersaline alkaline waters [12]. This outside fish supply for ospreys relies on the presence of rainbow trout, (*Oncorhynchus mykiss*) and cutthroat trout (*Oncorhynchus clarkia*) stocked in the streams and nearby lakes by the California Department of Fish & Wildlife with the goal of supplementing local fish populations to support fishing and human consumption [12, 14]. Fish stocked come from hatchery farms located all along north and central California.

Due to their predatory feeding habits, raptors have been a subject of interest for the study of comparative gastrointestinal anatomy and physiology among birds [15]. Important differences in gastrointestinal length, size, presence of crop, ceca length and size, among others have been identified for different taxa among this polyphyletic group of predatory birds [15]. Being a specialized fish eater, numerous studies have reported hunting behavior and success, type, and frequency of different fish species in osprey’s diet [4]. Ospreys are unique birds of prey due to their specialized feeding and foraging ecology that is accompanied by unique physiological and anatomical features [2]. However, studies on the gastrointestinal physiology, including gastrointestinal microbiota composition and the presence of antimicrobial resistant bacteria in this specialist raptor are scarce [16]. Several published reports have highlighted the importance of gastrointestinal microbiota in animal species, including birds [17, 18]. These studies on raptor’s microbiome have been focused on scavengers [19–21], on mammal eaters [16] and even on highly specialized species such as honey buzzards (*Pernis apivorus*) [22] and lesser kestrels (*Falco naumanni*) [23], in most of these cases by investigating birds admitted to rehabilitation centers [16]. Studies on wild raptors are relatively uncommon [16]. Furthermore, the presence of enteropathogens that may show antimicrobial resistance have been demonstrated by isolation and/or molecular characterization of antibiotic-resistant bacteria in the respiratory and gastrointestinal system of several species of raptors worldwide [24–27]. However, there is a paucity of studies conducted on Ospreys. In the only study where antimicrobial resistance was investigated for ospreys, multi-drug-resistant *Escherichia* (*E*.) *coli* and tetracycline-resistant *Enterococcus* (*E*.) *faecalis* were not isolated [25]. The aim of the present study was to investigate the presence of Enterobacterales, including isolation of β-lactamase-producing determinants and *Salmonella* spp., *Enterococcus* spp., *Staphylococcus* spp., and *Mammaliicoccus* spp. in the cloaca of nestling osprey population of Mono Lake, California, U.S.A. Furthermore, molecular characterization, antimicrobial and biocidal susceptibility testing was performed on the isolated bacteria.

## Material and methods

### Ethics statement

Capture, handling, and sampling of nestling ospreys were done by Peter H. Bloom, Ashli Lewis, and Miguel D. Saggese under permits provided by the California Department of Fish and Wildlife (Permit #S-190320004-19036-001) and the United States Fish and Wildlife Service (Permit #20431) and approved by the Western University of Health Sciences Institutional Animal Care and Use Committee.

### Study area and animal samples

Mono Lake is located in Mono County, central western California (38.0128° N, 118.9762° W). Its origin dates up to around one million years ago, in a high desert basin located in the Eastern Sierra Nevada Mountain range. Its endorheic waters are filled by water from rainfall and tributary streams. The highly alkaline waters do not support many life forms, except shrimp and flies, but no fish are found there [28]. The high levels of calcium carbonate can precipitate in these waters creating underwater formations locally known as tufas which can reach several meters of height above the water in and on the periphery of the lake as result of a decrease in water levels. For a detailed description of Mono Lake see [29]. For additional information on tufas formation see [30].

As result of the monitoring efforts started by the California State Parks on this population of ospreys, since 2000, nestlings produced on this lake were banded. On July 13, 2021, nests were accessed by boat between 7:00 AM-2:00 PM. Nests were climbed using a ladder to confirm content, and when present, to take nestlings down for sampling and banding. On each nest site, nests were accessed and inspected for content. Nestling ospreys were gently removed from their nests and temporarily handled for physical examination and sample collection using classical raptor handling techniques [31]. Nestlings were identified with color, alphanumeric, bands on their legs. Upon physical examination, cloacal swabs were taken by carefully introducing a cotton swab in the cloaca of these birds, gently rotated for a few seconds, and saved in Amies Transport Medium (Copan Diagnostics Inc, Murrieta, CA). Swabs were kept refrigerated at 4°C until arrival to the laboratory for bacterial isolation, with the exception during the flight between USA and Austria.

### Bacterial isolates

The isolation of bacteria, antimicrobial susceptibility testing, and molecular characterization with the exception of whole genome sequencing were performed at the University of Veterinary Medicine Vienna. For isolation of Enterobacterales, each sample was preincubated at 37°C overnight in buffered peptone water (BPW) (Merck, Germany). An aliquot of 200 μL of the incubated sample was cultured at 37°C overnight in BD™ MacConkey Broth (BD, Heidelberg, Germany) and then cultivated at 37°C overnight on BD™ MacConkey II Agar (Becton Dickinson (BD), Heidelberg, Germany). Selective isolation of β-lactamase-producing Enterobacterales and selective isolation of *Salmonella* spp. was performed as described previously (Grünzweil et al., 2021). The isolation of *Staphylococcus* spp. and *Mammaliicoccus* spp. was conducted by incubating each sample at 37°C overnight in tryptic soy broth (BD, Heidelberg, Germany) with 6.5% (w/v) NaCl and subsequently streaked onto BD™ Columbia CNA Agar with 5% Sheep Blood, Improved II (BD, Heidelberg, Germany). In addition, the isolation of methicillin-resistant staphylococci and mammaliicocci was achieved as recently described (Schauer et al., 2021). For the isolation of *Enterococcus* spp., each sample was incubated at 37°C in BBL™ Enterococcosel™ Broth (BD, Heidelberg, Germany) and then streaked onto BBL™ Enterococcosel™ Agar (BD, Heidelberg, Germany) and incubated at 37°C for 24 h. The respective colonies of isolates of interest showed the typical colony appearance for the abovementioned bacteria and were identified to the species level by matrix-assisted laser desorption ionization-time-of-flight mass spectrometry (MALDI-TOF MS) (Bruker Daltonik, Bremen, Germany) were saved at -80°C for further analyses.

### Antimicrobial and biocide susceptibility testing

Biocide susceptibility testing was performed at was performed at Department of Functional Food Products Development, Faculty of Biotechnology and Food Science, Wroclaw University of Environmental and Life Sciences. All susceptibility tests were conducted by agar disk-diffusion method according to CLSI, 2021 standards [32]. For Enterobacterales, the following antimicrobial agents (all from Becton Dickinson, Heidelberg, Germany) were used: ampicillin (10 μg), cefotaxime (30 μg), ceftazidime (30 μg), cefoxitin (30 μg), aztreonam (30 μg), imipenem (10 μg), meropenem (10 μg), gentamicin (10 μg), tobramycin (10 μg), amikacin (30 μg), ciprofloxacin (5 μg), trimethoprim-sulfamethoxazole (1.25/23.75 μg), tetracycline (30 μg), doxycycline (30 μg), chloramphenicol (30 μg), and fosfomycin (200 μg). Susceptibility testing of staphylococci and mammaliicocci was performed with the following antimicrobial agents: penicillin (10 units), cefoxitin (30 μg), ciprofloxacin (5 μg), amikacin (30 μg), gentamicin (10 μg), tetracycline (30 μg), erythromycin (15 μg), clindamycin (2 μg), chloramphenicol (30 μg), trimethoprim-sulfamethoxazole (1.25/23.75 μg), nitrofurantoin (300 μg), rifampicin (5 μg), and linezolid (30 μg). Penicillin (10 units), vancomycin (30 μg), teicoplanin (30 μg), ciprofloxacin (5 μg), tetracycline (30 μg), erythromycin (15 μg), chloramphenicol (30 μg), nitrofurantoin (300 μg), rifampicin (5 μg), linezolid (30 μg) were used for susceptibility testing of enterococci. Staphylococcal interpretative criteria have also been applied for mammaliicocci. *E*. *coli* ATCC^®^ 25922 and *Staphylococcus* (*S*.) *aureus* ATCC^®^ 25923 served as the quality control strains. AmpC-hyperproducing isolates (stably de-repressed) of *Citrobacter* spp. were examined as previously described [33].

Biocide susceptibility testing was performed according to the previously established protocol [34]. Benzalkonium chloride (Acros Organics, Geel, Belgium, 21541), representing the quaternary ammonium compounds, was tested at concentration ranges 0.000015%–0.016%, chlorhexidine (Sigma-Aldrich, Schnelldorf, Germany, 55-56-1), representing cationic compounds, was tested at concentration ranges 0.000015%–0.002%, glutardialdehyde (Chempur, Piekary Slaskie, Poland, 424610240), representing aldehydes, was tested at concentration ranges 0.0075%–1%, and isopropanol (99.9%, PHPU Eurochem BGD, Tarnow, Poland), representing alcohols, was tested at concentration ranges 1–14%. The method was performed in 96-wells U-bottom polystyrene microtiter plates (Sarstedt, Numbrecht, Germany, 82.1582.001). The bacterial inoculum was prepared as previously described [34] using Trypticasein soy broth (Biomaxima, Lublin, Poland, PS 23–500). The final concentration of bacteria inoculated into the wells was 2.5–5 × 10^5^ CFU/mL.

### Molecular identification and typing methods

Species identification of staphylococci and mammaliicocci was performed by *rpoB* sequencing [35]. The revisited Clermont method determined the phylogroup of the *E*. *coli* isolates [36]. Clonal relatedness of *E*. *coli* isolates was assessed by two-locus sequence typing of combined data of *fumC* and *fimH* sequences, as described previously [37], using CHTyper [38] hosted at the Center for Genomic Epidemiology (https://cge.cbs.dtu.dk/services/chtyper/, accessed on 1. April 2024), as well as *Escherichia/Shigella* database hosted on EnteroBase (https://enterobase.warwick.ac.uk/species/index/ecoli) [39]. Clonal relatedness of *Mammaliicoccus* (*M*.) *sciuri* isolates was characterized by MLST as previously described [40]. Briefly, internal segments of the seven house-keeping genes *ack*, *aroE*, *ftsZ*, *glpK*, *gmk*, *pta1* and *tpiA* were amplified. The PCR reactions were performed starting with 94°C for 300 s, followed by 30 cycles of 30 s at 94°C, 30 s at the respective annealing temperatures (55°C except for ftsZ (53°C)), 1 min at 72°C and a final extension at 72°C for 5 min. The amplicons were further purified and amplicons were Sanger sequenced with the same primer pair used for amplification at LGC Genomics Berlin, Germany. Allelic numbers and profiles were assigned using the MLST database hosted on PubMLST (https://pubmlst.org/organisms/mammaliicoccus-sciuri, accessed on 1. April 2024) [41]. The *spa* typing of *S*. *pseudintermedius* was performed as described previously [42].

### Virulence-associated genes

Detection and analysis of virulence-associated genes of *E*. *coli* isolates was performed using custom-made microarrays from INTER-ARRAY (INTER-ARRAY by fzmb GmbH, Bad Langensalza, Germany) according to manufacturer’s instructions [33, 43]. For the complete list of virulence-associated genes analyzed see S1 Table.

### Whole-Genome Sequencing (WGS)

Whole genome sequencing was performed at Austrian Agency for Health and Food Safety, Institute of Medical Microbiology and Hygiene. Fifteen isolates (*E*. *coli* (n = 5), *Citrobacter* (*C*.) *braakii* (n = 2), *M*. *sciuri* (n = 2), *Edwardsiella* (*E*.) *alberti*, *E*. *faecalis*, *E*. *faecium*, *Klebsiella* (*K*.) *aerogenes*, *S*. *pseudintermedius* and *S*. *delphini* (each n = 1) were selected based on their resistance and/or virulence load to be analyzed by WGS. Bacterial DNA was isolated using the MagAttract HMW DNA Kit (Qiagen, Hilden, Germany). Ready-to-sequence libraries were prepared using Nextera XT DNA Library Preparation Kit (Illumina, San Diego, United States). Isolates were paired-end-sequenced using the Illumina MiSeq platform with a read length of 2 × 300 bp [44]. *De novo-*assembly of raw reads was performed using SPAdes v.3.9.0 [45], and WGS data analysis was performed with SeqSphere+ software (Ridom, Münster, Germany). The assembled contigs were analyzed in the JSpecies workspace using the ANIb (average nucleotide identity via BLAST) analysis tool [46]. To assess the genetic relatedness of the examined isolates, classical MLST data were extracted from the corresponding database. Sequence types (ST) of *E*. *coli* and *E*. *albertii* were assigned using the *Escherichia/Shigella* database hosted on EnteroBase. STs of *C*. *braakii*, *K*. *aerogenes*, *M*. *sciuri*, *E*. *faecalis*, *E*. *faecium*, and *S*. *pseudintermedius* were determined using databases hosted at PubMLST (*C*. *braakii*: https://pubmlst.org/organisms/citrobacter-spp, *K*. *aerogenes*: https://pubmlst.org/organisms/klebsiella-aerogenes, *M*. *sciuri*: https://pubmlst.org/organisms/mammaliicoccus-sciuri, *Ent*. *faecalis*: https://pubmlst.org/organisms/enterococcus-faecalis, *Ent*. *faecium*: https://pubmlst.org/organisms/enterococcus-faecium, and *S*. *pseudintermedius*: https://pubmlst.org/organisms/staphylococcus-pseudintermedius) [41]. In addition, *E*. *coli* phylotypes were extracted from WGS by Clermont Typing (http://clermontyping.iame-research.center/, accessed on 1. April 2024) [47, 48]. CH types were characterized as mentioned above, and serogenotypes were analyzed by SerotypeFinder (https://cge.cbs.dtu.dk/services/SerotypeFinder/, accessed on 1. April 2024) [49]. To identify acquired resistance genes and/or chromosomal mutations, the Comprehensive Antibiotic Resistance Database (CARD; https://card.mcmaster.ca/home, accessed on 1. April 2024) [50], as well as ResFinder 4.1 (https://cge.cbs.dtu.dk/services/ResFinder/, accessed on 1. April 2024) [51], were used. Genes associated with biocide resistance were compared with the BacMet database (Antibacterial Biocide and Metal Resistance Genes Database, http://bacmet.biomedicine.gu.se/, accessed on 1. April 2024) [52]. Virulence genes were identified using VirulenceFinder 2.0 (https://cge.cbs.dtu.dk/services/VirulenceFinder/, accessed on 1. April 2024) [53] as well as the Virulence Factor Database (VFDB; http://www.mgc.ac.cn/VFs/, v) [54]. The presence of plasmids was assessed using PlasmidFinder 2.1 (https://cge.cbs.dtu.dk/services/PlasmidFinder/, accessed on 1. April 2024) [55]. Probability prediction of the location of a given *bla* resistance gene in Enterobacterales was achieved by applying mlplasmids trained on *E*. *coli* or *Klebsiella* [56]. Posterior probability scores (ppp) >0.7 and a minimum contig length of 1000 bp indicate that a given contig is plasmid-derived. For genes with ppp scores below 0.7, BLAST searches were performed for the respective contig sequence. If the BLAST search listed only plasmids first with 100% coverage and identities, a plasmid location was assumed. Gene prediction and annotation were performed using Genome Annotation Service PATRIC [57]. The genomes of WGS isolates were deposited under PRJNA1047016 in the NCBI BioProject database.

## Results

### Birds

A total of 18 nestling Ospreys, from ten different nests were banded and sampled. Age ranged from 3 to 6.5 weeks. All birds were found in overall good physical condition, alert, and responsive to handling.

### Bacterial isolates

In total, 44 isolates from 18 cloacal samples were obtained. The most commonly identified species was *E*. *coli* (n = 13), followed by *M*. *sciuri* (n = 6), *S*. *delphini* (n = 5), *E*. *faecalis* (n = 5), *E*. *faecium* (n = 4), *Hafnia alvei* (n = 2), *Klebsiella* (*K*.) *pneumoniae* (n = 2), *C*. *braakii* (n = 2), and singletons *E*. *tarda*, *E*. *albertii*, *K*. *aerogenes*, *Plesiomonas* (*P*.) *shigelloides* and *S*. *pseudintermedius*.

### Antimicrobial and biocide susceptibility testing and detection of resistance genes

Ten out of 13 *E*. *coli* isolates were susceptible to all tested antimicrobial agents. The remaining *E*. *coli* isolates (20-1a, 21-ctx and 22c-ctx) displayed an ESBL phenotype and two of them (21-ctx and 22c-ctx) were multi-drug resistant (MDR) [58]. Isolate 20 1a/P0 was resistant to β-lactam antibiotics ampicillin, amoxicillin/clavulanic acid and cefotaxime and carried *bla*_EC_. The gene *bla*_CTX-M-55_ was observed in both multi-drug resistant isolates. In addition, the detection of non-β-lactamase genes reflected well the phenotypic resistance profiles of the respective isolates. Thus, the isolate 21ctx that was resistant to tetracycline, chloramphenicol, trimethoprim-sulfamethoxazole and ciprofloxacin carried *tet*(A), *floR*, *sul3*, *dfrA14*, and the analysis of the quinolone resistance-determining regions (QRDR) revealed mutations in *gyrA*, *parC* and *parE*. Besides ampicillin, amoxycillin/clavulanic acid, cefotaxime and ceftazidime, the second *bla*_CTX-M-55_ positive isolate (22c-ctx/P8) was also resistant to gentamicin, tobramycin and chloramphenicol and harbored corresponding *aac(3)-IIa* and *floR* resistance genes (Table 1). Among other Enterobacterales isolates, resistance to 3^rd^ generation of cephalosporins (cefotaxime and ceftazidime) was observed in *K*. *aerogenes* isolate 22b ctx, which displayed both, an AmpC and ESBL phenotype. In addition, the *K*. *aerogenes* isolate was resistant to fosfomycin. WGS revealed that this isolate harbored *ampC* (EC 3.5.2.6) β-lactamase, *ampH* (encodes β-lactam binding protein AmpH) and *fosA5* genes. *C*. *braakii* isolate 22a-ctx was identified as a stably de-repressed AmpC-producer (Table 1). The mlplasmids analyses could not clearly predict whether the *bla*_CTX-M-55_ gene, which is identical in both isolates, is plasmid-encoded (ppp< 0.7). However, BLAST analyses of the respective contigs identified exclusively *E*. *coli* plasmids with 100% identity over the full contig length (4.27 and 5.83 kb, respectively). Another approach for an in-silico plasmid assembly from WGS data (see Virulence-assoc. Genes) also indicates the *bla*_CTX-M-55_ gene to be encoded on a large plasmid. Gene *bla*_EC-1149_ BLDB (http://bldb.eu; last accessed 1. April 2024 [59] detected in *E*. *coli* isolate 20 1a is predicted as chromosome-encoded (ppp 0.055 0.945) as well as *ampC* (EC 3.5.2.6) detected in *K*. *aerogenes* isolate 22b ctx (ppp 0.008). Two out of six *M*. *sciuri* isolates were resistant to clindamycin solely (*sal*(A)). Among staphylococci, the *S*. *pseudintermedius* isolate 19a was resistant to penicillin (*blaZ*). Resistance to rifampicin was observed in four *E*. *faecalis* isolates. *E*. *faecalis* isolate 17b as additionally resistant to tetracycline. WGS analysis revealed that this isolate carried the gene *tet*(M), and no known mutation in gene *rpoB*. Rifampicin resistance was also observed in three *E*. *faecium*.

**Table 1:** Summarized characterization of the Enterobacterales investigated: Genotype and resistance.

			Resistance									
ID*	Nest ID	Bacteria	Resistance**	Resistance genotype	Resistance associated mutation	ST***	*E*. *coli* phylogroup	*fumC*	*fimH*	Serotype	Biocide MIC (if elevated)*****	Biocide resistance genotype
6	ST08	*Plesiomonas shigelloides*	NR									
7	ST08	*Escherichia coli*	NR				E	36	93			
8	NB01	*Escherichia albertii*	NR			ST12692	chuAalbertii	111	644	nt		
9	NB01	*Escherichia coli*	NR				B1	19	86			
11, 12	LV09	*Escherichia coli*	NR				B1	1961	38			
13	LV02	*Escherichia coli*	NR				B1	19	32			
14	LV02	*Escherichia coli*	NR			ST20	B1	4	25	H2 O15		
17	ST15	*Escherichia coli*	NR				B2	24	9			
19	ST04	*Escherichia coli*	NR				B1	4	31			
19, 23	ST04, LV04	*Klebsiella pneumoniae*	NR									
20	LV04	*Escherichia coli*	NR				A	11	41			
20 1a	LV04	*Escherichia coli*	AMP, CTX	*bla*EC		ST1968	A	11	41	H11 O145		
21	OM02	*Edwardsiella tarda*	NR									
21 ctx	OM02	*Escherichia coli*	AMP, CTX, CAZ, CIP, TET, SXT, CHL	*bla*CTX-M-55, *bla*EC, *tet*(A), *floR*, *sul3*, *dfrA14*, *aadA1*, *aph(3’)-Ia*	parE:p.S458A, gyrA:p.D87N, gyrA:p.S83L, parC:p.S80I	ST361	A	99	54	H30 O45		
22a ctx	OM03	*Citrobacter braakii*		*bla*CMY-93		ST109						
22b ctx	OM03	*Klebsiella aerogenes*	CAZ, CTX, FOS	*bla*CMY, *fosA*5	ompK36 (N49S, T184P, A93S), ompK37 (I70M, I128M)	ST242						
22c ctx	OM03	*Escherichia coli*	AMP, CTX, CAZ, GEN, TOB, CHL	*bla*CTX-M-55, *aac(3)-IIa*, *aadA1*, *aph(3’)-Ia*, *floR*, *sul3*		ST58	B1	4	32	H25 O8		
22	OM03	*Hafnia alvei*	NR									
22e	OM03	*Citrobacter braakii*	NR	*bla*CMY-82		ST109						
23	LV04	*Hafnia alvei*	NR									
23 1a	LV04	*Escherichia coli*	NR			ST162	B1	65	32	H28 O8		

*—bacterial identification number

**—PEN, penicillin; AMP, ampicillin; CTX, cefotaxime; CAZ, ceftazidime; CIP, ciprofloxacin; TET, tetracycline; SXT, trimethoprim-sulfamethoxazole; CHL, chloramphenicol; FOS, fosfomycin; CLI, clyndamycin; RIF, rifampicin; NR, non-resistant

***—ST, sequence type

****—BAC, Benzalkonium Chloride; CHX, Chlorhexidine, ISO, Isopropanol

*E*. *faecium* isolate 22b/P8 was MDR to penicillin, ampicillin, ciprofloxacin, erythromycin, tetracycline (*tet*(S)) and rifampicin resistance (Table 2) and carried a hybrid-like *pbp5* gene of S8/R13-type 8 [60]. The same S/R-type is found in *E*. *faecium* strains EnGen35, EnGen21 and EnGen52 which show variable ampicillin MICs of 1, 8 and 128 ug/ml, respectively, so that also *pbp5* mRNA levels and PBP5 abundance could play a role.

**Table 2:** Summarized characterization of the staphylococci, mammaliicocci, and enterococci investigated: Genotype and resistance.

			Resistance						
ID*	Nest ID	Bacteria	Resistance**	Resistance genotype	Resistance associated mutation	ST***	*spa* type	Biocide MIC (if elevated)*****	Biocide resistance genotype
6, 7	ST08	*Mammaliicoccus sciuri*	NR			ST30			
8, 9, 10, 13, 19b	NB01 (8,9), LV09 (10), LV02 (13), ST04 (19b)	*Staphylococcus delphini*	NR						
9, 16	NB01, ST15	*Enterococcus faecalis*	RIF						
11	LV09	*Enterococcus faecalis*	NR					CHX (0,0005)	
11	LV09	*Mammaliicoccus sciuri*	NR			ST112			
13	LV02	*Enterococcus faecium*	NR						
14	LV02	*Mammaliicoccus sciuri*	CLI	*mecA1*, *sal*(A)		ST119			
15	LV02	*Mammaliicoccus sciuri*	CLI	*mecA1*, *sal*(A)		ST113			
17b	ST15	*Enterococcus faecalis*	TET, RIF	*tet*(M), *lsa*(A), *efrA*	gyrA:p.D759N	ST116			
18	ST04	*Mammaliicoccus sciuri*	NR			ST113			
18	ST04	*Enterococcus faecalis*	RIF					CHX (0,0005),	
								BAC (0,0005)	
19a	ST04	*Staphylococcus pseudintermedius*	PEN	*blaZ*		ST2199	t23		
20	LV04	*Enterococcus faecium*	RIF					BAC (0,0005)	
21	OM02	*Edwardsiella tarda*	NR						
22b	OM03	*Enterococcus faecium*	PEN, AMP, CIP, TET, ERY, RIF	*tet*(S), *msr*(C), *efmA*, *aac(6’)-Ii*	gyrA:p.N708D, AMP = Resistant pbp5 (p.A216S), pbp5 (p.S27G), pbp5 (p.L177I), pbp5 (p.R34Q), pbp5 (p.G66E), pbp5 (p.E100Q), pbp5 (p.V24A), pbp5 (p.N496K), pbp5 (p.T172A), pbp5 (p.T324A), pbp5 (p.E525D), pbp5 (p.K144Q), pbp5 (p.A499T)	ST355		BAC (0,0005)	*smr*
23	LV04	*Enterococcus faecium*	RIF					BAC (0,0005)	

*—bacterial identification number

**—PEN, penicillin; AMP, ampicillin; CTX, cefotaxime; CAZ, ceftazidime; CIP, ciprofloxacin; TET, tetracycline; SXT, trimethoprim-sulfamethoxazole; CHL, chloramphenicol; FOS, fosfomycin; CLI, clyndamycin; RIF, rifampicin; NR, non-resistant

***—ST, sequence type

****—BAC, Benzalkonium Chloride; CHX, Chlorhexidine, ISO, Isopropanol

The *E*. *faecium* isolate 22b, which had an elevated BAC MIC (0.0005), a gene coding for a small multi-drug resistance (SMR) transporter, which mediates resistance to quaternary ammonium compounds (QACs) was detected by analyzing WGS data (Table 2).

### Molecular typing methods

Most *E*. *coli* isolates were assigned to the group B1 (n = 8), followed by group A (n = 3), and singletons B2, whereas *E*. *albertii* belonged to chuAalbertii. Among *E*. *coli* isolates, the *fumC* and *fimH* (CH) clonotyping divided *E*. *coli* and *E*. *albertii* isolates into 12 distinct CH clonotypes. Two isolates, 11/M1 and 12/ML shared the same CH clonotype (CH1961-38). The same applies for isolates 20/P0 and 20-1a/P0 (CH11-41). All other *E*. *coli* isolates were singletons. WGS based serogenotyping disclosed all analyzed *E*. *coli* isolates into distinct serogenotypes: O15:H2, O145:H11, O45:H30, O8:H25 and O8:H28. WGS based MLST (Achtman 7 loci MLST) detected five different STs among the five sequenced *E*. *coli* isolates: ST20, ST58, ST162, ST361 and ST1968, whereas the *E*. *albertii* isolate was assigned to the new ST12692. Both *C*. *braakii* isolates (22a ctx; 22e) belonged to ST109 and the *K*. *aerogenes* isolate 22b ctx to the new ST242. Among six examined *M*. *sciuri*, four STs were detected: ST30 (n = 2) and three new ST112, ST113 (n = 2) and ST119. Sequence types of single *E*. *faecalis* (ST116), *E*. *faecium* (ST355), and *S*. *pseudintermedius* (new ST2199) were extracted from WGS data (Table 2). The *S*. *pseudintermedius* isolate belonged to *spa* type t23. PlasmidFinder identified seven different replicon types IncFIB(AP001918), IncFIC(FII), IncI1-I(Alpha), p0111, IncFII, ColpVC and IncFIA among *E*. *coli isolates*. IncFIB(AP001918) was detected in *E*. *albertii*. Finally, repUS43 replicon was identified in *E*. *faecalis*.

### Characterization of virulence-associated genes

In all eight *E*. *coli* isolates screened by the DNA microarray-based method, the adhesion gene *fimH* was detected. In addition, *E*. *coli* isolate 9 carried *cnf1* gene. WGS analysis of virulence-associated genes associated with *E*. *coli* isolates predicted the combination of *fimH*, *papC* and *iucD* characterizing the uropathogenic strains (UPEC) pathotype in the *bla*_CTX-M-55_-positive isolate (22c-ctx). The presence of *eae* and the absence of the *bfp* operon (encoding the bundle forming pilus—BFP) was detected in *E*. *coli* isolate 14 as well in *E*. *albertii* isolate 8 characterizing an atypical enteropathogenic *E*. *coli* (aEPEC). WGS-based analysis of virulence-associated genes of other selected bacteria revealed various virulence traits in all examined genomes (Tables 3 and 4).

**Table 3:** Summarized characterization of the isolates investigated–virulence Enterobacterales.

ID*	Nest ID	Bacteria	Virulence**
7	ST08	*Escherichia coli*	*fimH*
8	NB01	*Escherichia albertii*	*hcpA*, *hcpB*, *hcpC*, *eae*, *faeD*, *faeE*, *faeF*, *faeH*, *faeI*, *papC*, *paa*, *fimA*, *fimB*, *fimC*, *fimD*, *fimE*, *fimF*, *fimG*, *fimH*, *fimI*, *cah*, *ehaA*, *ehaB*, *upaG*, *vat*, *ibeB*, *ibeC*, *chuA*, *chuS*, *chuT*, *chuU*, *chuW*, *chuX*, *chuY*, *espB*, *espF*, *espG*, *map*, *tir*, *espK*, *espM2*, *espN*, *espW*, *espX6*. *espX7*, *nleC*, *aec15*, *aec16*, *aec17*, *aec18*, *aec19*, *aec22*, *aec23*, *aec24*, *aec25*, *aec26*, *aec27*, *aec28*, *aec29*, *aec30*, *aec31*, *aec32*, *cesT*, *escC*, *escF*, *escI*, *escJ*, *escN*, *escO*, *escR*, *escS*, *escT*, *escU*, *escV*, *espA*, *espD*, *etgA*, *glrR*, *ler*, *sepD*, *sepL*, *sepQ*, *cdtB*
9	NB01	*Escherichia coli*	*cnf1*, *fimH*
11	LV09	*Escherichia coli*	*fimH*
12	LV09	*Escherichia coli*	*fimH*
13	LV02	*Escherichia coli*	*fimH*, *(hlyA)*
14	LV02	*Escherichia coli*	*cfaA*, *cfaB*, *cfaC*, *cfaD/cfaE*, *ecspA*, *espB*, *ecpC*, *espD*, *ecpE*, *ecpR*, *elfA*, *elfC*, *elfD*, *elfG*, *eaeH*, *hcpA*, *hcpB*, *hcpC*, *eae*, *faeC*, *faeD*, *faeE*, *faeF*, *faeH*, *faeI*,*toxB*, *fimA*, *fimB*, *fimC*, *fimD*, *fimE*, *fimF*, *fimG*, *fimH*, *fimI*, *agn43*, *ehaA*, *ehaB*, *upaG*, *ibeB*, *ibeC*, *espB*, *espG*, *map*, *tir*, *espL1*, *espL2*, *espL4*, *espR1*, *espW*, *espX1*, *espX4*, *espX5*, *nleA*, *nleB1*, *nleH1-1*, *aec15*, *aec16*, *aec17*, *aec18*, *aec19*, *aec22*, *aec23*, *aec24*, *aec25*, *aec26*, *aec27*, *aec28*, *aec29*, *aec30*, *aec31*, *aec32*, *cesD2*, *cesT*, *escC*, *escD*, *escF*, *escJ*, *escN*, *escO*, *escR*, *escS*, *escT*, *escU*, *escV*, *espA*, *espD*, *ler*, *sepL*, *sepQ*, *hlyE*
17	ST15	*Escherichia coli*	*fimH*
19	ST04	*Escherichia coli*	*fimH*
20	LV04	*Escherichia coli*	*fimH*
20 1a	LV04	*Escherichia coli*	*ecpA*, *ecpB*, *ecpC*, *ecpD*, *ecpE*, *elfA*, *elfC*, *elfD*, *elfG*, *hcpA*, *hcpB*, *hcpC*, *fimA*, *fimB*, *fimC*, *fimD*, *fimE*, *fimF*, *fimG*, *fimH*, *fimI*, *aatA*, *ehaB*, *eaeX*, *ibeB*, *ibeC*, *espL1*, *espL4*, *espX4*, *espX5*, *espY1*, *aec15*, *hlyE*
21 ctx	OM02	*Escherichia coli*	*aafC*, *afaB*, *cfaA*, *cfaB*, *cfaC*, *cfaD/E*, *ecpA*, *ecpB*, *ecpC*, *ecpD*, *ecpE*, *elfA*, *elfC*, *elfD*, *elfG*, *eaeH*, *hcpA*, *hcpB*, *hcpC*, *fimA*, *fimC*, *fimD*, *fimE*, *fimF*, *fimG*, *fimH*, *fimI*, *pilQ*, *pilR*, *pilS*, *pilV*, *pilW*, *agn43*, *cah*, *ehaB*, *ibeB*, *ibeC*, *iucA*, *iucB*, *iucC*, *iucD*, *iutA*, *sitA*, *sitB*, *sitC*, *sitD*, *espL1*, *espL4*, *espR1*, *espX1*, *espX4*, *espX5*, *aec15*, *aec17*, *aec18*, *aec19*, *aec22*, *aec23*, *aec24*, *aec25*, *aec26*, *aec29*, *aec30*, *aec31*, *aec32*, *hlyE*,
22b ctx	OM03	*Klebsiella aerogenes*	*mrkH*, *mrkI*, *rimA*, *fimC*, *fimD*, *fimE*, *fimF*, *fimG*, *fimH*, *fimI*, *fimK*, *acrA*, *acrB*, *iutA*, *entA*, *entB*, *entC*, *entE*, *entF*, *entS*, *fepA*, *fepB*, *fepC*, *fepD*, *fepG*, *fes*, *iroB*, *iroC*, *iroD*, *iroE*, *iroN*, *rcsB*, *clpV/tssH*, *hcp/tssD*, *impA/tssA*, *sciN/tssJ*, *tssF*, *tssG*, *vasE/tssK*, *vgrG/tssI*, *vipA/tssB*, *vipB/tssC*, *clpV*, *dotU*, *icmF*, *ompA*, *impA*, *impF*
22c ctx	OM03	*Escherichia coli*	*aafC*, *afaB*, *cfaA*, *cfaB*, *cfaC*, *cfaD/E*, *ecpA*, *ecpB*, *ecpC*, *ecpD*, *ecpE*, *elfC*, *elfD*, *elfG*, *eaeH*, *hcpA*, *hcpB*, *hcpC*, *papC*, *papC*, *papI*, *papK*, *fimA*, *fimC*, *fimD*, *fimE*, *fimF*, *fimG*, *fimH*, *agn43*, *cdiA*, *ehaB*, *upaG*, *ibeB*, *ibeC*, *tia*, *iucA*, *iucB*, *iucC*, *iucD*, *iutA*, *sitA*, *sitB*, *sitC*, *sitD*, *fyuA*, *irp1*, *irp2*, *ybtA*, *ybtE*, *ybtP*, *ybtQ*, *ybtS*, *ybtT*, *ybtU*, *ybtX*, *espL1*, *espL4*, *aec15*, *aec16*, *aec17*, *aec18*, *aec19*, *aec22*, *aec23*, *aec24*, *aec25*, *aec26*, *aec27*, *aec28*, *aec29*, *aec30*, *aec31*, *aec32*, *hlyE*
23 1a	LV04	*Escherichia coli*	*cfaA*, *cfaB*, *cfaC*, *cfaD/cfaE*, *ecpA*, *ecpB*, *ecpC*, *ecpD*, *ecpE*, *elfA*, *elfC*, *elfD*, *elfG*, *hcpA*, *hcpB*, *hcpC*, *fimA*, *fimC*, *fimC*, *fimE*, *fimF*, *fimG*, *fimH*, *fimI*, *ehaB*, *upaG*, *vat*, *ibeB*, *ibeC*, *iucA*, *iucB*, *iucC*, *iucD*, *iutA*, *sitA*, *sitB*, *sitC*, *sitC*, *iroB*, *iroC*, *iroD*, *iroE*, *iroN*, *espL1*, *espL4*, *espR1*, *espX1*, *espX4*, *espX5*, *aec15*, *aec16*, *aec17*, *aec18*, *aec19*, *aec22*, *aec23*, *aec24*, *aec25*, *aec26*, *aec27*, *aec28*, *aec29*, *aec30*, *aec31*, *aec32*, *hlyE*

*—bacterial identification number

**—obtained by DNA microarray or identified using VFDB

**Table 4:** Summarized characterization of the isolates investigated–virulence staphylococci, mammaliicocci, and enterococci.

ID*	Bacteria	Virulence**
8	*Staphylococcus delphini*	*fnbB*, *icaA*, *icaB*, *icaC*, *sdrD*, *sdrE*, *geh*, *lip*, *nuc*, *adsA*, *sec*, *lukD*, *lukE*
14	*Mammaliicoccus sciuri*	*clfB*, *icaA*, *icaB*, *icaC*, *lip*, *sspA*
15	*Mammaliicoccus sciuri*	*icaA*, *icaB*, *icaC*, *sspA*
17b	*Enterococcus faecalis*	*asa1*, *ace*, *ebpA*, *ebpB*, *ebpC*, *srtC*, *efaA*, *cpsA*, *cpsB*, *cpsD*, *cpsF*, *cpsH*, *cpsJ*, *cpsK*, *bopD*, *fsrA*, *fsrB*, *gelE*, *sprE*
19a	*Staphylococcus pseudintermedius*	*clfB*, *fnbB*, *sdrE*, *geh*, *lip*, *nuc*, *adsA*, *galE*, *sec*, *lukE*
22b	*Enterococcus faecium*	*ebpA*, *ebpB*, *ebpC*, *srtC*, *ecbA*, *efaA*, *cpsA*, *bopD*

*—bacterial identification number

**—identified using VFDB

Analysis of WGS of *E*. *coli* isolate 22c ctx by VirulenceFinder detected several virulence traits like *iutA*/*iucA*, *sitA*, *iss*, *ompT* and *hlyF*, which are generally associated with virulence of avian pathogenic *E*. *coli* (APEC). When comparing the WGS data with plasmids from APEC strains using GeneiousPrime, more than 55% of the 174,240-bp plasmid pAPEC-O1-ColBM was encoded by contigs of strain 22c-ctx. Moreover, the aerobactin siderophore system (*iutA*/*iucABCD*), the iron/manganese transport system *sitABCD*, the increased serum survival gene *iss*, the same RepFIIA and RepFIC-like replicons reported from pAPEC-O1-ColBM, as well as the Colicin B and Colicin M region, all genes were located on contigs which were categorized as highly plasmid-associated rated by mlplasmids (ppp>0.7).

A BLAST search of the concatenated plasmid-predicted contigs of *E*. *coli* 22c-ctx/P8 identified multiple >100-kb *Enterobacteriaceae* plasmids. When using the best-listed pTREC1 of *E*. *coli* as a backbone for alignment, over 90% of the 128,358-bp plasmid could be reconstructed by WGS contigs of both *E*. *coli* isolates, 22c-ctx/P8 and 21ctx/P7. Interestingly, this also placed an AMR island on the predicted plasmid, consisting of *sul3*, *bla*_CTX-M-55_, *aadA*, *qacL*, *aac(3)-IIe* and *aph(3´)-Ia*.

## Discussion

In the present study, a population of ospreys at Mono Lake, California, was investigated for the presence of Enterobacterales, *Staphylococcus* spp., *Mammaliicoccus* spp., and *Enterococcus* spp. with special consideration of their antimicrobial resistance profile and their virulence-associated genes, which was analyzed by microarray analysis and WGS.

To the best of the authors’ knowledge, this is the first detailed investigation on the molecular characterization, virulence, and antimicrobial and biocide susceptibility of selected bacteria isolated from the cloacae of nestling ospreys, and from ospreys nesting in Mono Lake, California, USA. Ospreys, at very early age in life, appear to harbor a wide range of bacteria in their gastrointestinal tract, as reflected by the cloacal specimens examined here. Little is known about the establishment of raptors’ gastrointestinal microbiome since hatching to fledging. This study indicates that even at 3 to 6.5 weeks of age, ospreys, specialized fish-eating birds of prey, are exposed to at least several species of Enterobacteria and Gram-positive cocci. Some species, like *C*. *braakii*, a member of *Citrobacter freundii* complex and *P*. *shigelloides* are ubiquitous bacteria found in water, fish, birds, and other vertebrates, including humans [61, 62]. Prey or water seems to be a likely source of these bacteria for ospreys [63]

Enterobacteria are commonly found in fish [64], being the water used in aquaculture potentially contaminated with pathogens [63]. However, the presence of multi-drug resistant isolates in Enterobacterales, such as *E*. *coli*, *K*. *aerogenes*, and Gram-positive cocci such as *E*. *faecium*, suggest an anthropogenic source (spill over), either from human waste or more likely by the use of antimicrobial agents in fish farming that has led to the emergence of resistant strains.

Mono Lake is an endorreic lake that receives the tributary waters of many creeks originated at >3,000 m in the Sierra Nevada, such as Lee Vining creek, Rush creek and Mill creek. Domestic animals, wild animals and human activities occur around them. These creeks can be a potential source of microorganisms and being a potential source of these and other bacteria for ospreys. The physical and chemical properties of Mono Lake support the presence of several Proteobacteria, although Enterobacterales have not been reported in previous studies on the microbial composition of this endorreic lake [65, 66].

Prey, in this case stocking fish, appear to be a more likely candidate as the source of these microorganisms for ospreys. Fish are stocked on many rivers, creeks, and lakes of California with the purpose of supplying water bodies for fishing activities. Ospreys nesting in Mono Lake prey upon fish found in rivers, creeks and hatcheries located far from their nesting sites in Mono Lake. Presence of wildlife, livestock, and human waste are potential sources of bacteria in lakes [65], fish [67] and may be the source of these microorganisms for Ospreys.

Among the 44 isolates characterized, four isolates displayed multi-drug resistance pheno- and genotypes (*E*. *coli* (n = 2), *K*. *aerogenes* and *E*. *faecium*). Both multi-drug resistant *E*. *coli* isolates exhibited an ESBL phenotype, carried *bla*_CTX-M-55_ and belonged to ST58 and ST361. The *bla*_CTX-M-55_ gene was first discovered in ESBL-producing *E*. *coli* and *K*. *pneumoniae* in Thailand in 2006 [68]. The *bla*_CTX-M-55_ gene belongs to the *bla*_CTX-M-1_ group and carries an A77V substitution relative to *bla*_CTX-M-15_. The *bla*_CTX-M-55_ gene is common in Enterobacterales in the Asian continent, especially in *E*. *coli* of human and animal origins [69]. Since the first detection, *bla*_CTX-M-55_ has been identified in Enterobacterales of human and animal (including wildlife) origin as well from environmental samples from around the globe [70]. *E*. *coli* ST58 is a pandemic clone isolated from humans and animal hosts [71]. *E*. *coli* ST58 belongs to the clonal complex (CC) 151, and it has been recognized mainly as uropathogenic *E*. *coli* (UPEC), and as such, it belongs to extraintestinal pathogenic *E*. *coli* (ExPEC). [71] recently analyzed a genome collection of 752 whole-genome sequences of *E*. *coli* ST58 isolates that originated from different hosts, including 93 originating from wild animals. In that study, a large ST58 sub-lineage was identified that is characterized by the near-ubiquitous carriage of ColV plasmids, which carry virulence-associated genes and genes typical of the Yersiniabactin High Pathogenicity Island [72]. Several genes were highly associated with this sub-lineage: *ugd* and *galF*, both encode enzymes involved in outer membrane lipopolysaccharide biosynthesis, two prophage integrase *intA* genes, the *mlrA* gene, a regulator of curli biosynthesis and biofilm formation and *fyuA*, a marker gene for Yersiniabactin High Pathogenicity Island (HPI) [71]. All genes except *intA* were detected in our isolate 22c ctx.

When performing a genome similarity search using PATRIC, the two closest homologs to the Osprey isolate 22c ctx were two human isolates, Ec 908541 (917/1000 K-mer counts), an ExPEC isolate from a human UTI patient, and Ec UPEC-203 (914/1000 K-mer counts), both with an ST58 background. Interestingly, among UPEC strains, the ST58 sequence type is rather rare, the PubMLST listed only five out of 592 UPEC strains as of ST58.

The APEC pathotype is associated with large plasmids, such as the 124-kb plasmid pAPEC-O103-ColBM, which carries virulence gene clusters of the ColV pathogenicity island and occasionally a multi-drug-resistant (MDR) island [73, 74]. ColBM and ColV plasmids seem to contribute to avian colibacillosis but also to septicemias, neonatal meningitis and UTI infections in humans [75]. Historically, the first ColBM plasmids were isolated from human UPEC isolates. Nowadays, it is speculated that ColBM plasmids may have evolved from ColV plasmids [73]. Even though *E*. *coli* isolates belonging to ST361 were isolated from different hosts including humans and carried different resistance genes (https://enterobase.warwick.ac.uk/species/index/ecoli, last accessed 1. April 2024), the combination of ST361 and *bla*_CTX-M-55_ was rarely observed. Three ST361 strains carrying *bla*_CTX-M-55_ were recently isolated from sheep in the USA [76]. ESBL *E*. *coli* belonging to phylogroup A and ST361 have already been detected in wild animals (wolf (*Canis lupus*)) in Portugal but harbored *bla*_CTX-M-32_ [77]. The combination of ST361 and *bla*_CTX-M-55_ has not been observed in wildlife yet (https://enterobase.warwick.ac.uk/species/index/ecoli, last accessed 1. April 2024). Besides two multi-drug resistant *E*. *coli* isolates carrying *bla*_CTX-M-55_, isolate 20 1a displayed an ESBL phenotype and harbored *bla*_EC-1149_, belonging to phylogroup A and ST1968. The *bla*_EC_ family class belongs to C β-lactamases (cephalosporinases) and were termed as extended-spectrum AmpC β-lactamases (ESAC) [78], which are chromosome-borne [79]. *E*. *coli* isolates carrying ESAC are rarely reported from animals [80], and were not associated with ospreys yet. ST1968 has nineteen entries in the Enterobase *Escherichia/Shigella* Database (https://enterobase.warwick.ac.uk/species/index/ecoli, last accessed 1. April 2024), of which none was associated with wildlife or carry *bla*_EC_ gene. Even though two *Escherichia* isolates were non-resistant, they are of particular interest due to their composition of virulence gened. *E*. *albertii* isolate ID 8 E5 with the new sequence type ST12692 and chuAlbertii phylogroup and *E*. *coli* isolate ID 14 M4 belonging to ST20-B1 harbored among other an *eae* gene, one of the most highly polymorphic genes of the locus of enterocyte effacement island (LEE), which encodes the adhesin intimin [81]. LEE has the ability to induce intestinal histopathology termed as attaching and effacing’ (A/E), which is characteristic for Enteropathogenic *E*. *coli* (EPEC) infections. Based on the absence of the *bfp* operon (encoding the bundle forming pilus—BFP), both strains are characterized as atypical EPEC (aEPEC) [82]. aEPEC isolates of *E*. *coli* and *E*. *albertii* are associated with human diarrhea worldwide and were isolated from domesticated and wild animals, but were not associated with colonization or infection in our sampled ospreys [83–86].

Among other Enterobacterales, *K*. *aerogenes* isolate ID 22b ctx belonged to new ST242 and displayed a multi-drug resistant phenotype. Interestingly, this isolate displayed both an AmpC (due to intrinsic cephalosporinase) as well as an ESBL phenotype. None of β-lactamases were detected. The alterations in major porins of OmpK36 were detected. The altered OmpK36 could be responsible either for a decrease of porin expression or a loss of porin that could lead in the MIC-increase of β-lactams [87, 88]. Antimicrobial-resistant *K*. *aerogenes* is rarely reported to be associated with wildlife. Very recently, *K*. *aerogenes* was isolated from *Syncerus caffer* and *Gorilla gorilla gorilla* from Gabon, but these isolates did not display acquired resistance [89]. There are no entries in the *K*. *aerogenes* PubMLST database associated with wildlife (https://pubmlst.org/organisms/klebsiella-aerogenes, last accessed 1. April 2024).

Besides the isolation of multi-drug and antimicrobial-resistant Enterobacterales, the isolation of multi-drug resistant isolates among Gram-positive bacteria, is an important finding. *E*. *faecium* isolate 22b/P8 that belonged to ST355 displayed a multi-drug-resistance phenotype. There are four entries of ST355 in the *E*. *faecium* PubMLST database (https://pubmlst.org/organisms/enterococcus-faecium, last accessed 1. April 2024) with no known host. ST355 is a double locus variant of CC17, which is a major human clonal lineage [90]. Reports on antimicrobial resistance in *E*. *faecium* isolated from wild animals and especially associated with the birds of prey are scarce. Multi-drug resistant *E*. *faecium* were isolated in various wild animals, including wild boars (*Sus scrofa*) [91] in Portugal, wolves (*Canis lupus*) in Italy [92], from different wild birds in Poland [93], wild rabbits from Portugal and Tunisia [94–96], buzzards (*Buteo buteo*) from Portugal [97], Iberian wolf (*Canis lupus signatus*) [98] and wild birds of Azores Archipelago [99]. *E*. *faecium* isolates that were not susceptible to at least one antimicrobial agent in one or two classes of antimicrobial agents could be also detected among wild animals. The majority of *E*. *faecium* isolates observed in those studies were resistant to tetracyclines and macrolides (reviewed in [90]). Ampicillin resistance detected in 22b ST355 as well as tetracycline resistance mediated by *tet*(S) gene was rarely observed in *E*. *faecium* associated with wildlife (all above-mentioned references). The *tet*(S) gene was identified among various Gram-positive genera (i.e. *Enterococcus*, *Lactococcus*, *Lactobacillus*, *Staphylococcus*, *Streptococcus*) (https://faculty.washington.edu/marilynr/tetweb3.pdf). Contrary to *tet*(S), the *tet*(M) gene that was identified in 17 *E*. *faecalis* is the most frequent *tet* gene in *Enterococcus* [90]. 17 *E*. *faecalis* belonged to ST116. ST116 is an infrequent clone and has been associated with human clinical specimens, poultry, the environment, and very recently with wild Magellanic penguins (*Spheniscus magellanicus*) from Cidreira Beach, Brasil (https://pubmlst.org/organisms/enterococcus-faecalis, [100].

*S*. *pseudintermedius* and *S*. *delphini* are animal-associated staphylococci. Both species belong to *S*. *intermedius*-group and are commonly isolated from domesticated and wild animals [42, 101, 102]. *Mammaliiccoccus* genus, another animal-associated genus, was recently separated from *Staphylococcus* into a new genus [103]. Among staphylococci and mammaliicocci, the resistance was observed only to penicillin mediated by *blaZ-blaI-blaR1* operon in *S*. *pseudintermedius*, and to clindamycin mediated by *sal*(A) in *M*. *sciuri*. The *blaZ* gene was observed in many staphylococci and mammaliicocci from different animal species including wildlife [101]. The *sal*(A) gene confer resistance to lincosamides, pleuromutilines, streptogramin A was first described in *M*. *sciuri* but later on also in *S*. *haemolyticus*, *S*. *epidermidis* and *S*. *xylosus* [101]. After performing MLST either by sequencing of seven loci or after extracting the data from genome assembly, all of the analyzed *M*. *sciuri* and S. *pseudintermedius* resulted in a new sequence type. This is interesting and deserves to be monitored in the future to elucidate whether this particular osprey population at Mono Lake harbored distinct clonal lineages of *S*. *pseudintermedius* and *M*. *sciuri*.

The principal limitations of our study reside in the low sample size (n = 18) and a single isolated population of ospreys investigated. A greater sample size would have provided a more diverse picture of the examined bacteria in nestling ospreys. In addition, a greater number of populations would have provided information on the geospatial variability of this globally distributed raptor. Another issue is that the samples originated from nestlings. Since a gastrointestinal microbiome is known to vary among nestling and adult raptors [104, 105], our results may not necessarily mirror the situation at different life stages nor for ospreys in other geographic locations. Even though trapping and sampling adults is a very difficult task due to the nature of ospreys, this deserves to be discussed in the future. However, our study provides basic data on the development of nestlings’ microbiota for this unique raptor species at an early life stage. The presence of virulent and AMR bacteria in nestlings of a raptor species could be a potential threat to them. Antibiotic exposure is a primary driver of AMR in bacteria. However, other factors released into the environment, such as heavy metals or biocides, can also contribute to the selection and emergence of AMR from wildlife [106]. Given the potential role of biocides in selecting for resistance, biocide susceptibility testing should be conducted in conjunction with antimicrobial susceptibility testing. Furthermore, analyzing the diversity of virulence genes offer valuable insights into the evolutionary factors that have influenced bacterial pathogenicity. Ultimately, studying bacteria from wildlife contributes to a deeper understanding of the interconnectedness of human, animal, and environmental health, as emphasized by the One Health approach.

Several isolates in this study may be considered virulent and potentially pathogenic for animals and humans. Exposure to these bacteria early in life when the immune system is not fully developed might become a source of primary or secondary infection for birds, other animals, and even humans. Strict following of guidelines for handling wild birds should be followed when handling this and other raptor species. The present study contributes to the growing evidence that antimicrobial-resistant bacteria are currently part of the microbiome of wild animals, even at very early stages in life. Ospreys may be good biosentinels for the presence of these microorganisms and antibiotic resistance in the environment. Moreover, antimicrobial resistance is evolving and spreading easily via genes frequently encoded on mobile genetic elements among bacteria. Therefore, the present study’s results emphasize the need for a One Health approach to tackle the global antimicrobial resistance crisis. A longitudinal study is warranted to elucidate whether the presence of antibiotic-resistant bacteria in this population represents transient colonization or a persistent phenomenon.

## Supporting information

S1 Table The complete list of virulence-associated genes analyzed using DNA microarray-based technology. (XLSX)
